# A Liouville Property of Holomorphic Maps

**DOI:** 10.1155/2013/265752

**Published:** 2013-05-15

**Authors:** Chengjie Yu

**Affiliations:** Department of Mathematics, Shantou University, Shantou, Guangdong 515063, China

## Abstract

We prove a Liouville property of holomorphic maps from a complete Kähler manifold with nonnegative holomorphic bisectional curvature to a complete simply connected Kähler manifold with a certain assumption on the sectional curvature.

## 1. Introduction

 Liouville property is an interesting topic in analysis since Liouville found that a bounded holomorphic function on the complex plane must be a constant function. It has been studied by many geometers. For example, Yau [1] studied Liouville property of harmonic functions on complete Riemannian manifold with nonnegative Ricci curvature and Tam [2] studied Liouville property of harmonic maps. By Yau's Schwartz lemma [3], every holomorphic map from a complete Kähler manifold with nonnegative Ricci curvature, to a complete Kähler manifold with holomorphic bisectional curvature not greater than a negative constant is a constant map. This is a Liouville property for holomorphic maps.

In this paper, we prove the following Liouville property of holomorphic maps. This is, in some sense, a generalization of Yau's Liouville property for holomorphic maps.

**Theorem 1**

Let *M* be a complete Kähler manifold with nonnegative holomorphic bisectional curvature and *N* a complete simply connected Kähler manifold with sectional curvature ≤−*c*/(1+*r*)^2^, where *c* is some positive constant and *r* is the distance function of *N* to a fixed point *o*. Then, any holomorphic map from *M* to *N* must be constant.

Our strategy to prove this result is by first showing that there are plenty of bounded pluri-subharmonic functions on the target *N* which is guaranteed by the classical Hessian comparison. Then, by using Ni-Tam's [4] Liouville type theorem for plurisubharmonic functions on complete Kähler manifolds with nonnegative holomorphic bisectional curvature and noting that the pull back of a pluri-subharmonic function along a holomorphic map is still pluri-subharmonic, we obtain the conclusion. 

## 2. Proof of the Main Result

In order to show the existence of enough bounded plurisubharmonic functions on complete simply connected Kähler manifolds with negative quadratically decayed sectional curvature, we need the following technical lemma.

**Lemma 2**

For any positive constant *c*, there is a bounded function *f* ∈ *C* ^2^([0, *∞*)) such that

1. *f*(0) = 0 and *f*(*r*) > 0 for *r* > 0;
2. *f*′(*r*) > 0 for *r* ≥ 0;
3. *f*′′(*r*) + *c*/(1 + *r*)coth⁡(*cr*/(1 + *r*))*f*′(*r*) ≥ 0 for *r* ≥ 0.

**Proof**

Note that

(1) $$\begin{array}{c} c\coth\left(\frac{cr}{1+r}\right)\to c\coth c \end{array}$$

as *r* → *∞* and *c*coth⁡*c* > 1. There are two positive numbers *R* and *δ*, such that

(2) $$\begin{array}{c} \frac{c}{1+r}\coth\left(\frac{cr}{1+r}\right)>\frac{1+\delta }{1+r} \end{array}$$

for any *r* ≥ *R*. Let

(3) $$\begin{array}{c} h\left(r\right)=\min\left\{\frac{1+\delta }{1+r},\frac{c}{1+r}\coth\left(\frac{cr}{1+r}\right)\right\}. \end{array}$$

Then, it is clear that *h* ∈ *C*([0, *∞*)). Moreover,

(4) $$\begin{array}{c} h\left(r\right)=\frac{1+\delta }{1+r} \end{array}$$

when *r* ≥ *R*. Let

(5) $$\begin{array}{c} f\left(r\right)=\int_{0}^{r}e^{-\int_{0}^{s}h(t)dt}\;ds. \end{array}$$

Then, it is clear that *f* ∈ *C* ^2^([0, *∞*)) satisfying (1) and (2). Moreover, when *r* ≥ *R*,

(6) f(r)=∫0re−∫0Rh(t)dt−∫Rsh(t)dt ds=∫0re−∫0Rh(t)dt−∫Rs((1+δ)/(1+t))dt ds=(1+R)1+δe−∫0Rh(t)dt∫0r1(1+s)1+δds≤(1+R)1+δe−∫0Rh(t)dt∫0∞1(1+s)1+δds.

So, *f* is bounded.

Finally,

(7) $$\begin{array}{c} {\left(\log f^{\prime }\right)}^{\prime }\left(r\right)=-h\geq -\frac{c}{1+r}\coth\left(\frac{cr}{1+r}\right), \end{array}$$

which is equivalent to (3).

**Lemma 3**

Let (*M* ^*n*^, *g*) be a complete simply connected Kähler manifold with sectional curvature ≤−*c*/((1+*r*)^2^), where *c* is some positive constant and *r* is the distance function of *M* with respect to a fixed point *o* ∈ *M*. Then, for any point *p* ∈ *M*, there is a bounded continuous plurisubharmonic function *ϕ* such that *ϕ* > 0 on *M*∖{*p*} and *ϕ*(*p*) = 0.

**Proof**

Let *r*_*p*_ be the distance function to *p*. Then, it is clear that the sectional curvature of *M* is not greater than −*c*_1_/(1+*r*_*p*_)^2^, where *c*_1_ is some positive constant. By Hessian comparison, we have

(8) $$\begin{array}{c} D^{2}r_{p}\geq \frac{\sqrt{c_{1}}}{1+r_{p}}\coth\left(\frac{\sqrt{c_{1}}r_{p}}{1+r_{p}}\right)\left(g-dr_{p}⊗dr_{p}\right). \end{array}$$

Let $e_{1}=(1/\sqrt{2})(\nabla r_{p}-J\nabla r_{p})$ and *e*_1_, *e*_2_,…, *e*_*n*_ be a parallel unitary frame along geodesic rays emanating from *p*. Then

(9) $$\begin{array}{c} {\left(r_{p}\right)}_{1}=\frac{1}{\sqrt{2}} \end{array}$$

and (*r*_*p*_)_*α*_ = 0 for any *α* > 1.

By Lemma 2, let *f* be a bounded function in *C* ^2^([0, *∞*)) such that

1. *f*(0) = 0 and *f*(*r*) > 0 for *r* > 0;
2. *f*′(*r*) > 0 for *r* ≥ 0;
3. $f^{\prime \prime }(r)+(\sqrt{c_{1}}/(1+r))\coth(\sqrt{c_{1}}r/(1+r))f^{\prime }(r)\geq 0$ for *r* ≥ 0.

Let *ϕ*(*x*) = *f*(*r*_*p*_(*x*)). Then, by (8), we have

(10) (ϕαβ−)=(f′(rp)(rp)αβ−+f′′(rp)(rp)α(rp)β−)≥(f′(rp)c11+rpcoth⁡(c1rp1+rp)(gαβ−−(rp)α(rp)β−)+f′′(rp)(rp)α(rp)β−)=(12[c11+rpcoth⁡(c1rp1+rp)f′(rp)+f′′(rp)]00c11+rpcoth⁡(c1rp1+rp)f′(rp)In−1),

which is nonnegative. Hence, *ϕ* is a bounded continuous pluri-subharmonic function on *M* with *ϕ* > 0 on *M*∖{*p*} and *ϕ*(*p*) = 0.

We are now ready to prove Theorem 1.

**Proof of Theorem 1**

We proceed by contradiction. Let *f* : *M* → *N* be a nonconstant holomorphic map. Then, there are two points *p*, *q* in *M*, such that *f*(*p*) ≠ *f*(*q*). By Lemma 3, let *ϕ* be a bounded continuous pluri-subharmonic function on *N* such that *ϕ*(*f*(*p*)) = 0, *ϕ*(*f*(*q*)) > 0. Then, *ϕ*(*f*) is a bounded continuous pluri-subharmonic function on *M* that is nonconstant. This contradicts Theorem  3.2 in [4].

**Remark**

The unitary invariant metric on *ℂ* ^*n*^ constructed by Seshadri [5] has negative sectional curvature and sectional curvature ≤−1/(*r* ^2^log⁡⁡*r*) outside a compact subset. This means that the curvature decayed rate cannot be raised to be greater than 2 in Theorem 1.
